# Cryopreservation Induces Acetylation of Metabolism-Related Proteins in Boar Sperm

**DOI:** 10.3390/ijms241310983

**Published:** 2023-07-01

**Authors:** Malik Ahsan Ali, Ziyue Qin, Shan Dou, Anqi Huang, Yihan Wang, Xiang Yuan, Yan Zhang, Qingyong Ni, Rameesha Azmat, Changjun Zeng

**Affiliations:** 1 Key Laboratory of Livestock and Poultry Multi-Omics, Ministry of Agriculture and Rural Affairs, Sichuan Agricultural University, Chengdu 611130, China; malik364ahsan@hotmail.com (M.A.A.); qinziyue1234@163.com (Z.Q.); sonic123wang@163.com (Y.W.); yxmercury@163.com (X.Y.); yanzhang@sicau.edu.cn (Y.Z.); niqingyong@hotmail.com (Q.N.); 2 Farm Animal Genetic Resources Exploration and Innovation Key Laboratory of Sichuan Province, College of Animal Science and Technology, Sichuan Agricultural University, Chengdu 611130, China; 3 Department of Theriogenology, Faculty of Veterinary Science, University of Agriculture, Faisalabad 38000, Pakistan; 4 College of Life Science, Sichuan Agricultural University, Ya’an 625014, China; imdoushan@sina.com (S.D.); huangaqii@sicau.edu.cn (A.H.); 5 Department of Biochemistry, Faculty of Science and Technology, Government College Women University, Faisalabad 38000, Pakistan; razmat714@gmail.com

**Keywords:** boar, sperm cryopreservation, protein acetylation, energy metabolism

## Abstract

Cryodamage affects the normal physiological functions and survivability of boar sperm during cryopreservation. Lysine acetylation is thought to be an important regulatory mechanism in sperm functions. However, little is known about protein acetylation and its effects on cryotolerance or cryodamage in boar sperm. In this study, the characterization and protein acetylation dynamics of boar sperm during cryopreservation were determined using liquid chromatography–mass spectrometry (LC-MS). A total of 1440 proteins were identified out of 4705 modified proteins, and 2764 quantifiable sites were elucidated. Among the differentially modified sites, 1252 were found to be upregulated compared to 172 downregulated sites in fresh and frozen sperms. Gene ontology indicated that these differentially modified proteins are involved in metabolic processes and catalytic and antioxidant activities, which are involved in pyruvate metabolism, phosphorylation and lysine degradation. In addition, the present study demonstrated that the mRNA and protein expressions of SIRT5, IDH2, MDH2 and LDHC, associated with sperm quality parameters, are downregulated after cryopreservation. In conclusion, cryopreservation induces the acetylation and deacetylation of energy metabolism-related proteins, which may contribute to the post-thawed boar sperm quality parameters.

## 1. Introduction

Different sperm cryopreservation protocols are employed to maintain the viability and fertility of sperm in assisted reproductive technologies like artificial insemination (AI) [1]. AI is widely used with domestic animals because of the increasing demand for milk and meat [2,3]. However, the sperms of different species behave differently due to cellular composition; hence, vulnerability to cryodamage also differs. Currently, only 1% of pig AI is carried out with frozen (post-thawed) semen because of the lower conception and fecundity rates [4,5,6]. It is well-established that rapid temperature fluctuations and the formation of ice crystals lead to cryodamage in the sperm plasma membrane [7,8]. The plasma membrane of boar sperm contains a higher concentration of phospholipids compared to other mammals, which increases its vulnerability to cold shock and cryo-injuries [6,9,10]. Sperm DNA and mitochondria are also susceptible to cryodamage [11], which can be related to cryopreservation in terms of differentially expressed mRNA, piRNA and miRNA [12,13]. Despite decades of research on cryopreserving semen, no multipurpose semen additive has been discovered to prevent boar sperm from suffering cryodamage and increase the rate of AI, which would, in turn, result in increased conception and fecundity rates. Therefore, the underlying factors that regulate cryodamage in sperm also need to be elucidated.

Sperm proteins play crucial roles in the regulation of sperm function, viability and fertility [14]. However, changes in protein content and structure can occur due to repeated freezing and thawing, consequently affecting the motility, viability and fertility of sperm [15]. Cryopreservation does not only affect sperm viability and fertility but also leads to post-translational modifications (PTMs), including acetylation, glycosylation, succinylation, ubiquitination, methylation and sumoylation [16,17,18,19,20]. Several reports suggest that sperm cells are transcriptionally silent [21]. However, a multitude of data is available related to the abovementioned post-translational modifications (PTMs) of these cells [22]. These PTMs occur mainly due to the production of free radicals, which cause oxidative stress in the sperm cell and, hence, control the functional abilities and structural integrities of the sperm either directly or indirectly. Important energy metabolic processes, including pyruvate metabolism and the tricarboxylic acid cycle, have also been shown to be modified by differentially expressed proteins [23,24]. The PTMs of these proteins, especially acetylation, control the energy metabolism via IDH1, IDH2, GLUT1, MDH2, LDHC, SIRT5 and many other enzymes [25,26,27]. For instance, IDH2 activity has been found to decrease after acetylation, which is determined by its metabolic product, succinate [28,29]. Similarly, GLUT1, GAPDH and PKM2 acetylation resulted in reduced activity and, hence, affected glycolysis and the citric acid cycle [30,31]. However, the activities of STAT3 and PDC (both involved in energy metabolism) increased after acetylation [31].

Protein acetylation involves the transfer of a functional acetyl group to a molecule or compound, which may result in increased or decreased activity [32]. The aberrant expression of proteins and their differential acetylation patterns may affect the normal physiological functions of sperm [33]. Salehi et al. reported that epigenetic patterns, such as DNA methylation (DMNT), histone methylation and acetylation, were reduced in rooster sperm after cryopreservation [34]. The acetylation of boar sperm was altered by different concentrations of available glucose [35]. The acetylation of lysine in ε-amino acid is the most abundant PTM and is catalyzed by acetyltransferase and can be reversed by deacetylases [36]. Thus, lysine acetylation an important regulatory mechanism for sperm functions [35,37]. Therefore, we hypothesized that these acetylated PTMs of sperm may regulate cryoresistance/cryodamage, which affects fertility, motility and other parameters. Thus, in this study, we first determined the differentially expressed acetylated proteins between fresh and frozen-thawed sperm via acetyl proteomic analysis. Then, the mRNA and protein expressions of four metabolic proteins (IDH2, LDHC, MDH2 and SIRT5) were verified. Finally, the effects of the knockdown of SIRT5 on the acrosomal integrity, motility and mitochondrial membrane potential (MMP) of boar sperm were also evaluated.

## 2. Results

### 2.1. Differentially Expressed Acetylated Proteins and Modified Sites during Sperm Cryopreservation

**Figure 1:**
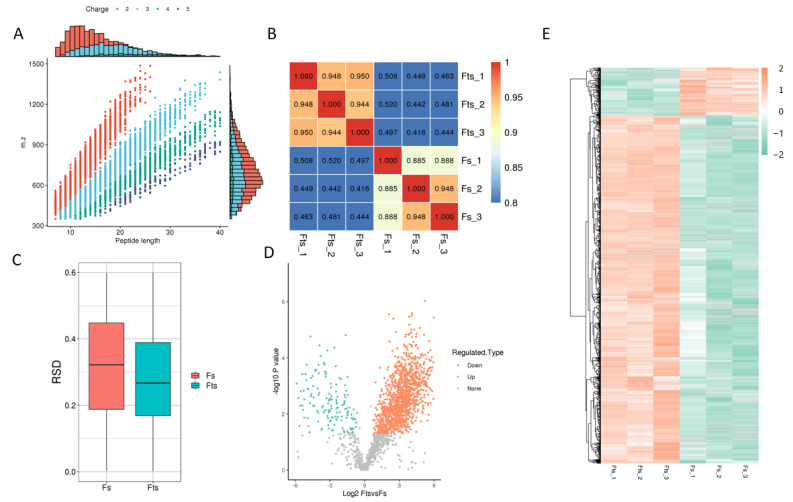
Identified proteins, peptides, sites and their correlation. Peptide length shows the number of amino acids present in different peptides (**A**); heat map is drawn using Pearson’s correlation coefficient (PCC): positive (close to +1), negative (close to −1) and no-correlation (close to 0) (**B**); boxplot shows the relative standard deviation between the fresh (Fs) and frozen (Fts) sperm: the smaller the overall relative standard deviation (RSD), the better the quantitative repeatability (**C**); volcano plot of the modified sites: orange and green dots represent the upregulated and downregulated modified proteins and sites, while grey indicated no change in expression (**D**); hierarchical diagram of the modified sites: the *X*-axis represents different fresh and frozen sperm groups and *Y*-axis represents the upregulated or downregulated proteins (**E**).

A total of 4567 peptides were found to be modified from 7640 identified peptides. However, the total number of protein spectra was 155,547, of which 1440 proteins were identified with 4705 modified acetylated sites (Table 1 and Figure 1A). The important proteins (top 10) that regulate the energy metabolism of sperm are listed in Table 2, along with their acetylation status in fresh and frozen (post-thawed) sperm. Additionally, a total of 1252 and 172 sites were upregulated and downregulated, respectively, in frozen and fresh sperm (fold change > 1.5). The obtained results are further elaborated in Figure 1. The peptides generated by enzymatic hydrolysis and mass spectrometry fragmentation showed that most of the peptides consisted of 7–20 amino acids (Figure 1A).

The heat map was plotted using Pearson’s correlation coefficient (Figure 1B) between pairs of all samples, and the degree of linear correlation between two data sets was taken as −1, +1 and 0, if it was close to negative, positive and uncorrelated, respectively. A boxplot plot of the RSD for protein quantification is shown in (Figure 1C), which indicates better repeatability in the case of a smaller overall RSD.

Moreover, protein modification sites were filtered, and differences between both comparison groups were evaluated by fold change (FC > 1.5). Data showed that 1251 and 171 sites were upregulated and downregulated, respectively. Similarly, 505 and 96 proteins were upregulated and downregulated, respectively. The volcano plot and heat map distributions of these proteins and sites are shown in Figure 1D,E.

### 2.2. GO and KEGG Analysis Indicating the Role of Modified Proteins in Energy Metabolism

**Figure 2:**
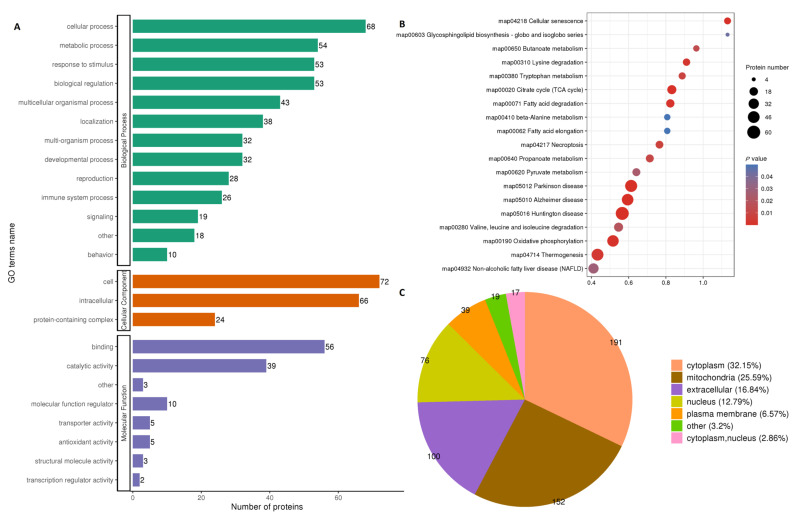
GO term analysis and Log2 fold change comparison between fresh and frozen–thawed groups. Gene ontology (GO) of proteins participating in biological functions (**A**); KEGG pathways regulated by modified proteins and sites (**B**); pie diagram showing subcellular localization of modified proteins and sites (**C**). All data are shown as Log2 fold change enrichment of the proteins for relative expression. The size of the circle in B indicates the number of proteins involved in a particular process along the *Y*-axis, while color intensity (strong red for higher and light blue for lesser roles) shows participation in different processes.

The differentially expressed proteins in both fresh and frozen sperms were found to regulate 474 biological processes (BP), 162 cellular components (CC) and 123 molecular functions (MF), according to the GO-term (Gene Ontology) analysis (Figure 2A). Most of the proteins were related to glycolytic, pyruvate metabolism, tricarboxylic acid cycle and fatty acid metabolism pathways. The detailed analysis of modified proteins included in the KEGG (Kyoto Encyclopedia of Genes and Genomes) pathways and subcellular pathways (pie diagram) are shown in Figure 2B,C, respectively. Mitochondrial pathways were found to be the second most abundant pathways after cytoplasmic pathways.

**Figure 3:**
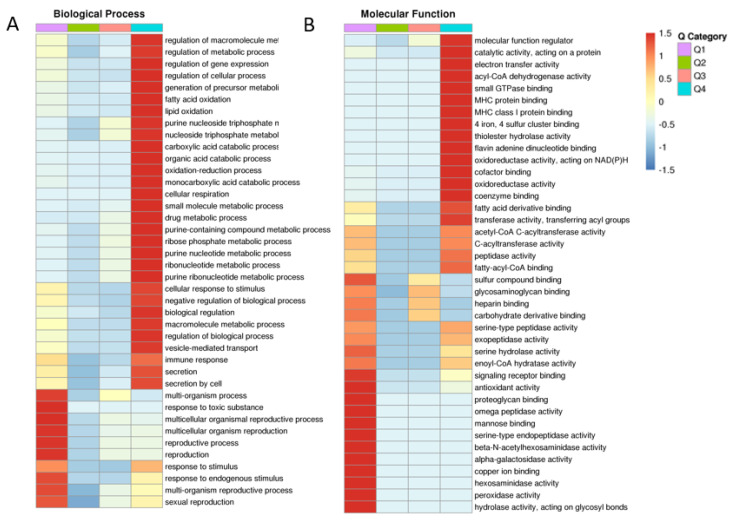
Cluster analysis showing the degree of enrichment of differentially expressed proteins in fresh and frozen sperm groups. Biological processes (**A**); molecular functions (**B**). The intense red color indicates the highest enrichment and blue indicates the lowest enrichment. Different groups (Q1, Q2, Q3 and Q4) and functions are given horizontally and vertically, respectively.

After determining BP, CC, MF and protein domain with GO, KEGG and protein domain enrichment analysis, fresh and frozen–thawed groups were divided into four parts, Q1–Q4, according to the differentially expressed sites (Figure 3). Important BP and CC components that were found to be enriched included metabolic processes, sexual reproduction, oxidoreductase and antioxidant activity. Cluster analysis was performed to find the correlation between differentially expressed proteins in the comparison groups (Figure 3A,B, for BP and MF; Supplementary Figure S1 for CC, KEGG and protein domain, respectively).

### 2.3. Protein–Protein Interaction

**Figure 4:**
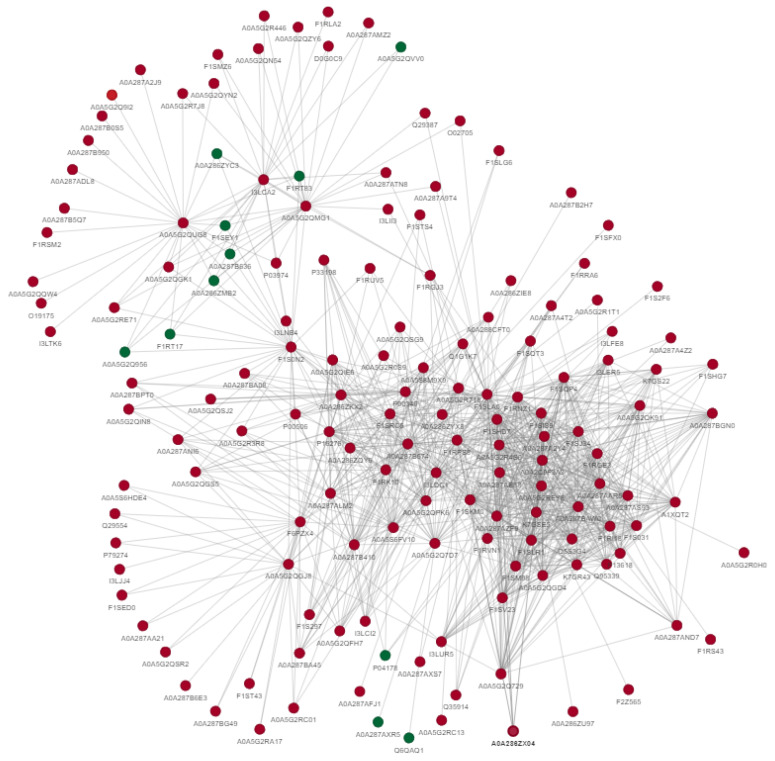
Protein–protein interactions of differentially acetylated proteins between fresh and post-thawed boar sperm. The green color indicates downregulated proteins, while red indicates upregulated proteins.

Protein–protein interactions indicate that differentially acetylated proteins are involved in the metabolism of carbohydrates, proteins, lipids and steroids. Furthermore, these proteins also regulate gene expression, phosphorylation, ubiquitination, MAPK-signaling, PI3k/AKT signaling, cellular structure and cellular responses to different stresses. The uniport IDs of these proteins are shown in the diagram depicting the interactions between these differentially expressed acetylated proteins (Figure 4).

### 2.4. mRNA and Protein Expression of IDH2, MDH2, LDHC and SIRT5

**Figure 5:**
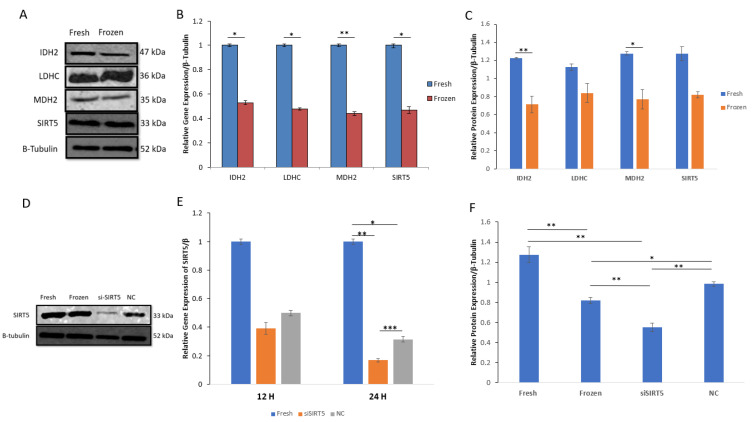
Relative mRNA and protein expressions of acetylated enzymes (IDH2, LDHC, MDH2 and SIRT5) in fresh and post-thawed boar sperm. (**A**) WB analysis indicates protein expression between fresh and frozen (post-thawed) sperms; (**B**) mRNA expression of IDH2, LDHC, MDH2 and SIRT5 between fresh and post-thawed sperms; (**C**) relative protein expressions of IDH2, LDHC, MDH2 and SIRT5 between fresh and post-thawed sperms; (**D**) WB analysis of SIRT5 in fresh, frozen, siSIRT5 and NC groups; (**E**) relative gene expression of SIRT5 in fresh, siSIRT5 and NC groups; (**F**) relative protein expression of SIRT5 in fresh, frozen, siSIRT5 and NC groups. All the data were subjected to statistical analysis and considered significant at (* *p* < 0.05) and highly significant at (** *p* < 0.01 and *** *p* < 0.001). Isocitrate dehydrogenase (IDH2); malate dehydrogenase (MDH2); sirtuin5 (SIRT5); lactate dehydrogenase (LDHC).

The important metabolic proteins involved in the tricarboxylic acid (TCA) cycle, including isocitrate dehydrogenase (IDH2), malate dehydrogenase (MDH2), lactate dehydrogenase (LDHC) and mitochondrial sirtuin5 (SIRT5), were evaluated in both fresh and post-thawed boar sperms. All the selected enzymes showed downregulation in post-thawed sperm at both the mRNA and protein levels. Moreover, the protein concentrations of IDH2 and MDH2 were significantly decreased (*p* < 0.01 and *p* < 0.05, respectively) in post-thawed sperm; however, LDHC and SIRT5 showed no significant decrease. Additionally, the mRNA expression of MDH2 was more significantly decreased (*p* < 0.01) compared to the mRNAs of other proteins (*p* < 0.05). The data for both mRNA and protein expressions are shown in Figure 5.

### 2.5. Knockdown of SIRT5 (siSIRT5) Affects Sperm Motility, Acrosomal Integrity and Mitochondrial Membrane Potential (MMP)

**Figure 6:**
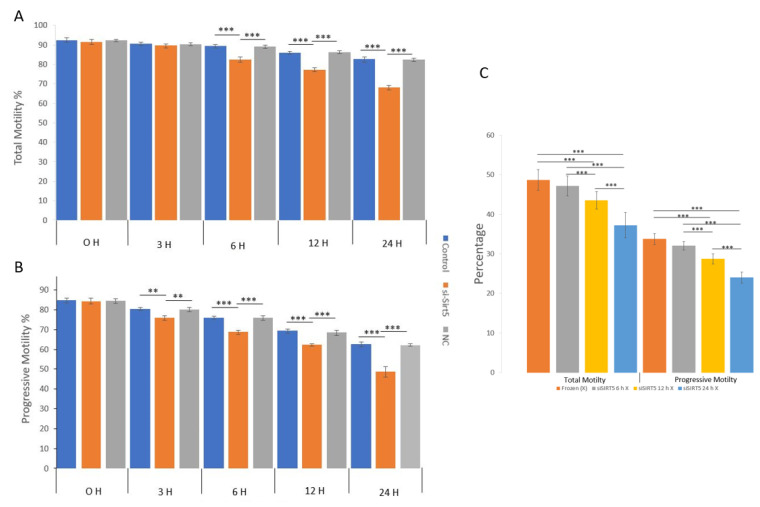
Knockdown of SIRT5 (siSIRT5) affects total and progressive sperm motilities. (**A**) Total and progressive motilities were significantly higher in the fresh group compared to the frozen (post-thawed) and siSIRT5-treated groups. Total motility was not significantly different among the fresh, siSIRT5 and NC groups before 6 h of transfection, but significant differences were observed at and after 6 h. (**B**) Progressive motility started to differ significantly at 3 h of transfection. (**C**) Total and progressive motilities were significantly higher in untreated frozen groups compared to siSIRT5-treated frozen groups. ** *p* < 0.01; *** *p* < 0.00.1. siSIRT5 6 h, siSIRT5 12 h X and siSIRT5 24 h X indicate transfection of sperm for 6 h, 12 h and 24 h, respectively, before freezing.

The total and progressive motilities of sperms were evaluated in the fresh, post-thawed, siSIRT5 and negative control (NC) treatment groups (Figure 6). The sperm total and progressive motilities showed significant (*p* < 0.001) differences in the fresh sperm group compared to the frozen and siSIRT5 treated groups after 6 h and 3 h of treatment, respectively. However, total sperm motility did not show any significant difference before 6 h of transfection with siSIRT5. Both the NC and siSIRT5 groups also showed significant differences (*p* < 0.001) in total (6 h) and progressive motilities (3 h). In addition, both motilities of siSIRT5-treated sperm were also significantly lower after 12 h and 24 h of transfection compared to the fresh and NC groups. However, no significant difference (*p* > 0.05) was observed between the control (fresh) and NC groups at any time intervals. Furthermore, both total and progressive motilities were compared between the frozen (without siSIRT5 treatment) and siSIRT5-treated frozen groups. Fresh sperm were transfected with siSIRT5 for 6 h (siSIRT5 6 h X) and 12 h (siSIRT5 12 h X) and then frozen to compare the motilities between these groups (Figure 6C).

**Figure 7:**
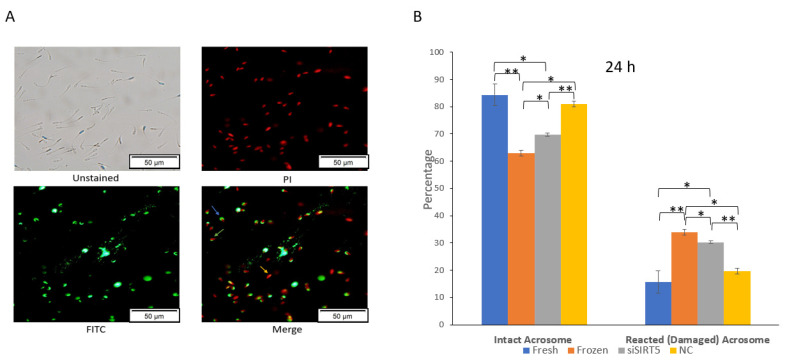
Knockdown of SIRT5 (siSIRT5) affects acrosomal integrity in boar sperm. (**A**) siSIRT5 sperms were subjected to PI and FITC-PNA stains: as shown in the image titled Merge, the red sperms (marked with a yellow arrow) are dead (PI^+^/FITC-PNA^−^); sperms with intact green caps have intact acrosome and membranes (blue arrow) (PI^+^/FITC-PNA^+^); sperms with damaged green caps are reacted ones (green arrow) (PI^+^/FITC-PNA^+^). (**B**) Acrosomal status was subjected to statistical analysis and evaluated after 24 h. The results are representative of at least three independent experiments (mean ± SEM). * *p* < 0.05; ** *p* < 0.01. Propidium iodide (PI); fluorescein isothiocyanate (FITC); peanut agglutinin (PNA); negative control (NC).

An intact acrosome is required for successful fertilization under in vitro or in vivo conditions. Cryopreservation causes an increase in the number of sperms with reacted (damaged) acrosomes, and these sperms cannot fertilize the oocyte (Figure 7 and Supplementary Figure S2). In this study, a significant (*p* < 0.05) decrease in sperms with intact acrosomes was observed in the frozen group compared to the fresh group after evaluation using a fluorescence microscope (Figure 7A). Similarly, the knockdown of SIRT5 also caused a significant (*p* < 0.05) decrease in the number of sperms with intact acrosomes after 24 h of siSIRT5 transfection compared to control sperms (fresh) (Figure 7B). Moreover, the difference between frozen (post-thawed) and siSIRT5-transfected sperm was also significant (*p* < 0.05). These data showed more damaged acrosomes in the frozen group compared to the siSIRT5 group.

**Figure 8:**
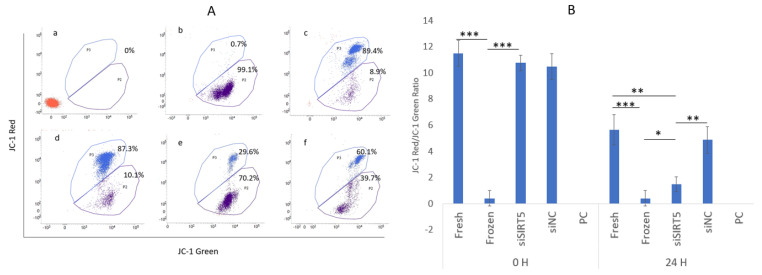
Mitochondrial membrane potential (MMP). Analysis of MMP by flow cytometry: blue color shows cells with higher MMP while purple indicates cells with lower MMP; control (without JC−1 dye), CCCP (positive control (PC)), cells with highly disrupted MMP), fresh (control), NC, frozen (post-thawed) and siSIRT5 treated groups are shown as a–f, respectively (**A**). The ratio of JC-1 red to green was sorted out to determine the statistical significance between the groups (**B**). * *p* < 0.05; ** *p* < 0.01; *** *p* < 0.001. Carbonyl cyanide 3-chlorophenylhydrazone (CCCP); negative control (NC).

The sperm mitochondrial membrane potentials (MMPs) were determined using JC-1 dye in control (without JC-1 dye), fresh (control), frozen (post-thawed), siSIRT5 and NC groups, while CCCP was used as a positive control (Figure 8). The results showed that MMP was significantly higher in the fresh group compared to the other groups (except for the NC) after 24 h of transfection (Figure 8A). A highly significant (*p* < 0.001) difference was observed between the fresh and frozen groups after 24 h of transfection. Interestingly, the transfected and frozen groups also showed a significant difference (*p* < 0.05) (Figure 8B). 

## 3. Discussion

Repeated freezing and thawing procedures lead to cryo-capacitation and apoptosis-like changes, which result in damaged plasma membranes, decreased acrosomal integrity, mitochondrial membrane potential and sperm energy metabolism, along with altered expressions of different genes, mRNAs and proteins [5,38,39,40]. Only a few studies have revealed the epigenetic modifications of these genes and their respective products that altered sperm survivability and fertility after cryopreservation [40,41]. These studies demonstrated that some genes, mRNAs and proteins play crucial roles by over- or under-expressing themselves in post-thawed sperms. In our previous studies, we deciphered that differential expressions of mRNA, miRNA, piRNA, odorant receptors and post-translational modifications (PTMs) of proteins regulate the energy metabolism, viability and fertility of sperm before and after cryopreservation in boars [6,12,18]. Methylation, as an important PTM, was found to regulate FOXO3, ACLY, HIF1A, NADK2, SLC9A3R1, FASN and PKM genes that control the quality of sperm in boars [18]. In boar sperm, the methylation levels of these genes were reported to be altered in fresh and frozen sperm, and only one FASN gene was hypomethylated compared to others that were hypermethylated. However, human studies of the LIT1, MEST SNRPN, MEG3 and H19 genes showed no change in methylation patterns [42]. Similarly, lysine and histone acetylation regulate spontaneous acrosomal reactions and energy metabolism in the sperm of bulls and boars [43,44]. Sperm glycosylation, ubiquitination and succinylation were found to contribute to sperm quality [44]. However, these PTMs have not been well compared between fresh and frozen sperms. In the current study, we reported that cryopreservation leads to protein acetylation, and differential expressions of IDH2, MDH2, LDHC and SIRT5 were observed between fresh and frozen sperm (post-thawed). To further elaborate on the role of differential expression of these mitochondrial enzymes in the regulation of sperm fertility parameters, SIRT5 was silenced using siRNA, and sperm motility, acrosomal integrity and mitochondrial membrane potentials were evaluated, which showed significant (*p* < 0.05) differences between siRNA-treated and untreated sperms.

Acetylated proteins regulate boar sperm energy parameters, which are essential to maintain the viability and fertility of sperm. MDH2 is an important protein in the TCA cycle, which controls the conversion of malate to oxaloacetate. In this study, MDH2 was downregulated after cryopreservation at both the mRNA and protein levels. Studies have shown that the altered regulation of MDH2 compromises energy metabolism, and its lysine acetylation results in increased activity [45,46]. However, in our study, the acetylation level of MDH2 increased in frozen sperm and its expression decreased at both the mRNA and protein levels. LDHC acetylation level is known to control different types of cancers like breast cancer, and testicular and colon cancer [47,48,49]. Its downregulation results in enhanced DNA damage and inhibition of DNA repair enzymes. Similarly, sperm analysis has shown that sperms with low LDHC expression have low conception and fertility rates compared to sperms with normal or enhanced expression [50]. In recent years, the acetylation of LDHC has been targeted for contraception and male fertility [37,51]. Similarly, IDH2 expression was downregulated in frozen sperm compared to fresh sperm, which shows consistency with previously published studies [52]. IDH2 is an important enzyme in the TCA cycle and is expressed in the mitochondria of sperms [53,54,55]. In one study, extensively used semen additives like bovine serum albumin and skim milk were found to regulate PTMs of boar sperm, which resulted in increased MMP, intracellular ATP contents and activity of GAPDH [44]. Similarly, the differential expressions of these proteins, along with other proteins, can be used as biomarkers to determine the fertility of sperm. For instance, PSP-1, PSP-2, spermine, SPADH1, SPADH2, spermadhesins, ENO1, GPX5, BSP1, BSP5, ACE and CRISP3, along with MDH2, LDHC and IDH2, regulate capacitation, litter size, motility, semen volume, fertility, preservation, morphology and maturation of sperm cells in boars and other animals [15,56,57,58,59,60,61,62,63,64,65]. Sperm motility and viability are directly linked to energy harboring and utilizing the capacity of available energy sources. Silent information regulator (sirtuins) is an important NAD+-dependent deacetylase family that controls metabolism, obesity, cancer and fertility [66]. Of this sirtuin family, SIRT3, SIRT4 and SIRT5 are abundantly expressed in the mitochondria and thus regulate energy metabolism [66]. SIRT5 regulates male fertility via the modulation of mitochondrial function and oxidative stress [67] by scavenging reactive oxygen species (ROS) produced during mitochondrial metabolism [68]. The acetylation or deacetylation of SIRT5 has not been studied and is linked to male fertility in mammals. In the current study, SIRT5 was found to be downregulated in frozen (post-thawed) sperm, and further investigation revealed its role in sperm motility, acrosomal integrity and MMP, which are important fertility parameters of male germ cells. The detailed exploration of SIRT5 in fresh and post-thawed sperm can be carried out in relation to boar fertility.

The PTMs related to histone modification and lysine acetylation mainly control the energy metabolism of cells [69,70]. These PTMs regulate glycolysis, the tricarboxylic acid (TCA) cycle and the electron transport chain, via which cells manufacture their energy in the form of ATP [69,71]. Enzymatic protein modifications of GLUT1, IDH2, MDH2, LDHC, SIRT5, PKM2 and PGAM1 regulate glycolysis and the TCA cycle [72,73,74]. Extracellular glucose is taken up by the cell with the activation of the GLUT1 enzyme, and hence the subsequent glycolysis occurs to provide cellular energy [75]. Similarly, previous studies have shown that differentially expressed and modified proteins due to cryopreservation are mainly involved in glycolysis, pyruvate metabolism, oxidative phosphorylation and the TCA cycle. In one of our previous studies, we elucidated that pyruvate dehydrogenase complex component X (PDHX) is more expressed in fresh boar sperm compared to post-thawed sperm, indicating the detrimental effect of cryopreservation on sperm energy metabolism [76]. This PDHX converts pyruvate into acetyl-CoA, which is an important link between glycolysis and the TCA cycle. Additionally, these modified proteins also regulate fatty acid and lysine degradation, along with participation in the cGMP-PKG pathway. This cGMP/PKG pathway regulates Ca^+2^ influx and tyrosine phosphorylation in the cell and thus helps achieve hyperactivation in the female reproductive tract [77], which is required for fertilization. The cGMP/PKG activation during cryopreservation leads to early capacitation of sperm cells, resulting in reduced motility, fertility and viability of sperm after artificial insemination [7,78].

Moreover, this study revealed that most of the modified proteins are mainly involved in biological processes, cellular components and molecular functions. The important processes and functions include metabolic processes (phosphorylation, lactate dehydrogenation, pyruvate metabolism), reproduction, cell signaling, catalytic activity, transporter activity, antioxidant activity and transcriptional regulatory activity. Moreover, protein domain analysis confirmed their roles in acyl-Co-A dehydrogenase C, middle and N-terminals. These acyl-Co-A dehydrogenase domain products regulate metabolic enzymes and are oxidized to provide energy [79]. Furthermore, the post-translational modification levels of these acetylated proteins are verified to control the proliferation and apoptosis of cells [80,81]. Out of these proteins, metabolically important proteins, i.e., IDH2, MDH2, LDHC and SIRT5, showed differential expression at the mRNA and protein levels in fresh and post-thawed sperm. In addition, SIRT5 was found to regulate the sperm fertility parameters, which can be considered as biomarkers, along with the expression of SIRT5 to verify fertility in boars and other species. Further scientific research on post-translational modifications, including acetylation, methylation and ubiquitination of different genes that control cell survival signals, metabolism and motility of boar sperm, may lead to increased success of conception and litter size.

## 4. Materials and Methods

### 4.1. Semen Collection and Processing

The gloved hand technique was used to collect fresh semen from 12 boars by strictly following the Administration of Affairs Concerning Experimental Animals (Ministry of Science and Technology, China, revised in June 2004) regulations, and the experiment was approved by the Institutional Animal Care and Use Committee of the College of Animal Science and Technology, Sichuan Agricultural University, Sichuan, China (under permit no. 2019202012).

Ejaculates from all 12 Landrace boars were collected after the initial evaluation of motility and fertility parameters, as described previously [76]. These ejaculates were divided into 3 groups, each containing semen from 4 animals. Each group of sperm was pooled together and further divided into 2 groups for fresh (Fs) and frozen (Fts) sperm experiments. Fresh sperms were marked as Fs1, Fs2 and Fs3 in all 3 respective groups. Similarly, Fts1, Fts2, and Fts3 were labeled to frozen sperm in all 3 respective groups. In addition, fresh sperm groups were further divided into siSIRT5 and negative control (NC) groups. Fresh sperms were directly subjected to protein extraction and subsequent analysis, whereas the frozen group was subjected to cryopreservation using our lab’s protocols [18]. Briefly, sperm centrifugation was carried out (at 1800 rpm for 5 min) and then a lactose egg yolk (LEY) extender containing egg yolk (10 mL) and 11% β-lactose (40 mL) was added. After semen extension, it was cooled to 4 °C at a rate of 0.2 °C decrease/min, and then further LEY was added to obtain a 3% glycerol concentration. Finally, semen was loaded into straws (FHK, Tokyo, Japan) and kept about 3 cm above the liquid nitrogen for 10 min and then plunged into it until further processing.

### 4.2. RNA Extraction and Quantitative PCR

RNA extractions from sperm cells were performed using the Trizol LS Reagent Kit (Invitrogen, Carlsbad, CA, USA) following our lab’s protocol [82]. After RNA extraction, only the samples with more than a 1.8 ratio of OD260/280 were selected for reverse transcription. Evo M-MLV RT mix kit (Code no. AG11728, Changsha, China) and SYBR Green Pro Taq HS qPCR kit (Code no. 11701, Changsha, China) were used for reverse transcription according to the manufacturer’s instructions. Finally, CFX96 Real-Time PCR Detection System (BioRad, Hercules, CA, USA) was used for qPCR for all three biological replicates. The results were quantified using the 2^−ΔΔCT^ method [83]. GAPDH was used as a reference gene and the primer sequences are listed in Table 3, along with the IDH2, MDH2, LDHC and SIRT5 primers.

### 4.3. Protein Extraction and Western Blotting

Sperm groups were subjected to protein extraction using RIPA lysis buffer (Beyotime Biotechnology, B0013B, Nantong, China). The sperm samples were transferred to Eppendorf tubes, and PBS was added to wash and separate sperm cells from their seminal fluid. After 2–3 washes with 1 mL PBS at 1500 rpm for 5 min each, PBS was completely removed by pipetting. Then, each sample was added to a lysis buffer (RIPA lysis buffer, Beyotime Biotechnology, B0013B, China) and centrifuged at 12,000 rpm for 15 min at 4 °C, following the manufacturer’s instructions. The supernatant was transferred to other Eppendorf tubes, and the BCA assay kit (Boster Biotechnology Co., Ltd., Pleasanton, CA, USA, AR0146) was used to determine the protein concentration according to the manufacturer’s instructions.

After protein extraction and concentration measurement, Western blot analysis was performed to determine the expression of proteins in fresh and frozen sperm. Western blotting was performed as described by [84] with a few modifications described briefly as follows: protein samples were mixed with Laemmle blue (containing 1 percent BME as a reducing agent) stain in a 3:1 ratio. After gentle mixing, the samples were placed at 95 °C for 5 min. Pre-cast electrophoresis gels were used to run the protein samples in the HEPES running buffer. The instrument settings were kept at 80 V for 20 min and then 120 V for 60 min. Upon completion of electrophoresis, the proteins were transferred to PVDF membranes (Beyotime Biotechnology, FFP32, China) using a transfer buffer at 400 mA for 40 min. After the transfer, the membranes were blocked with Quick Block Buffer for 15 min on a shaker. The membranes were then incubated with primary antibodies (Anti-IDH2 antibody 1:1000, # AB177512, Abcam, Shanghai, China; MDH2 antibody 1:1000, # 8610, Cell Signaling Technology, Shanghai, China; Anti-LDH-C mAB 1:1000, # 6302, PTM BIO; SIRT5 polyclonal antibody 1:2000, # PA5-31029, Invitrogen) overnight at 4 °C. All the antibodies were prepared using the Quick Block^TM^ primary antibody dilution buffer for Western blotting (Cat no. P0256, Beyotimes Biotechnology, Shanghai, China). After incubation, the membranes were washed with a wash buffer (Cat no. P0023C6, Beyotime, Shanghai, China) and then incubated again with a secondary antibody (Ultrapolymer Goat anti-Rabbit IgG (H & L) HRP, # PR30011, Proteintech, Wuhan, China) for 1 h. Finally, the membranes were washed again 3 times to remove the unconjugated secondary antibody. After completion of the procedure, the membranes were probed for protein bands on an eBlot Touch Imager (Shanghai, China) using the enhanced chemiluminescence (ECL) detection kit, BeyoEcl Moon kit (P0018FS, Beyotime Biotechnology, China). Band intensities were subjected to ImageJ software (v. 1.48) and quantified for at least three independent experiments.

### 4.4. Enzymatic Hydrolysis

Protein samples were hydrolyzed using trypsin (Sigma-Aldrich, St. Louis, MO, USA, T8658), an enzyme present in the gastrointestinal tract of mammals, to digest the protein. Extracted protein samples were mixed with 20% TCA and allowed to precipitate for 2 h at 4 °C. The mixture was then centrifuged at 4500 rpm for 5 min, and the supernatant was discarded. The precipitate was washed 2 times with pre-chilled acetone. The pellets were then dried and triethylammonium bicarbonate (TEAB) (Sigma-Aldrich, 241059) was added to obtain a final concentration of 200 mM. Trypsin was added at a ratio of 1:50 (protease:protein *m*/*m*) and allowed to hydrolyze overnight. After digestion with trypsin, dithiothreitol (DTT) (Sigma-Aldrich, 43815) was added to obtain a final concentration of 5 mM and then reduced for 30 min at 56 °C. Finally, iodoacetamide was added to obtain a final concentration of 11 mM and incubated for 15 min at room temperature after covering it with silver paper (to avoid light).

### 4.5. Modification Site Enrichment and Mass Spectrometry Analysis (LC-MS)

The peptides obtained after enzymatic hydrolysis were dissolved in IP buffer solution (100 mM NaCl, 1 mM EDTA, 50 mM Tris-HCl, 0.5% NP-40 and pH 8.0) (ThermoFisher Scientific, Waltham, MA, USA, catalog number: 87787), centrifuged at 10,000 rpm for 5 min, and the supernatant was transferred to a pre-washed acetylated resin (PTM-104, Hangzhou Jingjie Biotechnology Co., Ltd., Hangzhou, China, PTM Bio). It was then placed on a rotary shaker at 4 °C, shaken gently and incubated overnight. After incubation, the resin was washed 4 times with an IP buffer solution and deionized water. Finally, resin-bound peptides were eluted three times with 0.1% trifluoroacetic acid elute (Sigma-Aldrich, 302031), collected and lyophilized under a vacuum. After drying, the eluate was desalted and vacuum freeze-dried for liquid chromatography–mass spectrometry (LC-MS) analysis.

The peptides were dissolved in liquid chromatography (LC) mobile phase A (aqueous solution of 0.1% formic acid and 2% acetonitrile) and mobile phase B (aqueous solution of 0.1% formic acid and 90% acetonitrile) and separated using the EASY-nLC 1200 ultra-high performance liquid system (ThermoFisher Scientific, LC140). Subsequently, after running the samples in the liquid phase (gradient setting: 0–36 min, 9~25% B; 36–54 min, 25~35% B; 54–57 min, 35~80% B; 57–60 min, 80% B and flow rate maintained at 500 nL/min), the peptides were separated using an ultra-high performance liquid phase system, injected into the NSI ion source for ionization, and then analyzed using Q Exactive™ HF-X mass spectrometry. The settings of LC-MS were as follows: ion voltage 2.1 kV; primary mass spectrum scan range 350–1600 *m*/*z*; scan resolution 120,000; secondary mass spectrum scan range 100 *m*/*z*; scan resolution 15,000; automatic gain control (AGC) 1 × 10^5^; signal threshold 5 × 10^4^ ions/s, maximum injection time 100 ms and dynamic exclusion time of the tandem mass spectrum was set at 15 s to avoid repeated sweeps of precursor ions. Orbitrap (Q Exactive™ HF-X mass spectrometer) was used to detect peptide precursors and their fragments. The data-dependent acquisition (DDA) program was used for data acquisition after the primary scan.

The resulting MS/MS data were processed using the MaxQuant search engine (v.1.6.15.0). Tandem mass spectra were searched against the boar SwissProt database (20,422 entries) concatenated with the reverse decoy database. Trypsin/P was specified as the cleavage enzyme, allowing up to 2 missing cleavages. The mass tolerance for precursor ions was set to 20 ppm in the first search and 5 ppm in the main search, and the mass tolerance for fragment ions was set to 0.02 Da. Carbamidomethyl at Cys was specified as a fixed modification, and acetylation at the N-terminus of the protein and oxidation on Met were specified as variable modifications. FDR was adjusted to <1%.

### 4.6. Protein Interaction, Functional Enrichment and Cluster Analysis

Protein enrichment analysis was performed using gene ontology (GO), the Kyoto Encyclopedia of Genes and Genomes (KEGG), and the protein domains of the differentially expressed proteins in each group. After GO, KEGG and protein domain enrichment analyses of the differentially expressed proteins in both comparison groups, these were further divided into four parts, namely, Q1, Q2, Q3 and Q4. The differentially modified protein sequence was screened according to the difference of factor 1.5 in both fresh and frozen sperm groups and compared with the STRING (v.11.0) protein interaction network database, and protein interactions were determined according to confidence score > 0.7 (high confidence). The differentially modified protein interaction network was then visualized using the R package “networkD3” tool.

### 4.7. Electrotransfection and Detection of Boar Sperm Quality Parameters

Sperm electrotransfection was carried out following Zhang’s protocol [85]. Briefly, sperm concentration was reduced to 5 *×* 10^7^ mL^−^^1^ with Beltsville thawing solution, as described in our previous paper [76]. Elecrotrasnfection was performed with 25 nM of siSIRT5 and NC (recommended by the manufacturer, RiboBio, Guangzhou, China) using an electro cell manipulator (ECM-2001, BTX, Holliston, Harvard, MA, USA). The machine setting was adjusted to deliver 4 cycles (duration for each cycle was 100 μs) at 300 V. Transfected sperms were kept under the same conditions as fresh sperm for subsequent experiments. The efficiency of transfection was determined by the expression of SIRT5 at the mRNA and protein levels.

Sperm motility was assessed in fresh, post-thawed, si-SIRT5 and NC groups at 0, 3, 6, 12 and 24 h intervals. A computer-assisted semen analyzer (CASA, Minitube, Germany) was used to measure the motility of sperm. The stage of the CASA microscope was pre-heated to 37 °C, and glass slides (Minitube) were kept in this stage to warm them to 37 °C. A total of 4 μL of sperm sample was loaded onto these glass slides and left for a few seconds to allow the sperm to settle down. Then, sperm motility and concentration were measured. At least 5 fields were selected for each sample, and the results were obtained as the means of these selected fields. 

Acrosomal integrity was determined in the fresh, post-thawed, siSIRT5 and NC groups. Peanut agglutination (PNA) was used to determine acrosomal integrity. Briefly, sperm cells were washed in all the groups and then fixed for 10 min in paraformaldehyde. Sperm cells were then treated with FITC-PNA (Sigma Aldrich, Burlington, MA, USA) working solution at a concentration of 20 µg/mL for 20 min at 37 °C. Then, PI (1 µL) (Sigma Aldrich, USA) was added into this solution and kept at 37 °C for 5 min. After this, PBS solution was used to wash the samples three times, and then the acrosomal status was evaluated under an epifluorescence microscope (Olympus, Tokyo, Japan). At least 200 sperms were evaluated in each sample, and ImageJ software (v. 1.48) was used to determine the acrosomal status by red and green fluorescence.

Sperm MMP was determined by JC-1 (Solarbio Life Sciences, Beijing, China, Cat no. J8030) reagent, and a working solution of JC-1 was prepared by adding 3 µL of JC-1 stock solution to 747 µL of PBS. From this working solution, 100 µL was added to 100 µL of semen and incubated at 37 °C for 20 min. Then, this mixture was centrifuged at 4000 rpm for 5 min, and the supernatant was discarded. Sperm cells were washed 3 times with 500 µL PBS at 4000 rpm for 5 min each time. Then, 800 µL PBS was added and mixed thoroughly, and FACS flow cytometry was used to determine the MMP of the sperm.

### 4.8. Statistical Analysis

Data are shown as mean ± SEM in this study. Statistical differences were determined using SPSS (v. 20.0) with independent samples *t* test for two groups, where *p* < 0.05 was considered statistically significant. Relative gene expressions were quantified using the 2^−ΔΔCT^ method. A one-way analysis of variance (ANOVA) was used to analyze the differences among the different groups. Protein quantification was performed using the ImageJ (v. 1.48) software. Pearson’s square method was used to find the correlation between differentially expressed proteins. For repeatability, Pearson’s correlation coefficient (PCC), principal component analysis (PCA) and relative standard deviation (RSD) methods were used.

## 5. Conclusions

Cryopreservation results in differential expression and acetylation of proteins in sperm cells, and these differentially expressed proteins regulate cellular energy metabolism via different enzymes and signaling pathways, which were found to be involved in pyruvate metabolism, fatty acid metabolism and tyrosine phosphorylation. Further exploration of these proteins showed higher levels of protein site modifications in the sense of acetylation. So, acetylation, an important PTM, regulates the energy metabolism-related proteins and affects post-thawed sperm functions and fertility.

## Figures and Tables

**Table 1:** Summary of MS/MS spectrum database search analysis (localization probability > 0.75).

Total Spectrums	Matched Spectrums	Peptides	Modified Peptides	Identified Proteins	Quantifiable Proteins	Identified Sites	Quantifiable Sites
155,547	33,829	7640	4567	1440	977	4705	2764

**Table 2:** The top 10 boar sperm metabolic proteins and their acetylation due to cryopreservation.

Protein	Acetylation Sites	Fold Change (Fts/Fs)	Acetylation Status	Function
PC	999, 261, 79	33.147	Up	TCA cycle, metabolic pathways
ENO1	239, 335, 71, 228, 233, 420	5.487	Up	Glycolysis, metabolic pathways
PGAM2	100	0.807	Down	Glycolysis, metabolic pathways
GAPDHS	49, 286, 147, 130, 115, 124	12.66	Up	Glycolysis, spermatogenesis, metabolic pathway
SIRT5	50, 150, 161	11.483	Up	Metabolic pathways, oxidation–reduction pathways, TCA cycle
IDH2	241, 244, 124, 232, 168	13.745	Up	Carbohydrate metabolism, organic acid metabolism, TCA cycle
MDH2	185, 328, 329, 335, 78, 301	21.913	Up	Metabolic pathways, TCA cycle
LDHC	228, 232, 81, 224, 217	9.568	Up	Metabolic pathways
GPX4	58, 132, 145, 172	36.776	Up	Glutathione metabolism, metabolic pathways
GPD2	607, 95, 410, 198, 114	15.156	Up	Carbohydrate metabolism

**Table 3:** Primer information for RT-qPCR.

Gene	Sequence (5′-3′)	Tm (°C)	Size (bp)
GAPDH	F: ACCCAGAAGACTGTGGATGG R: CATGGCCTCCAAGGAGTAAG	60.07	346
IDH2	F: GGGCCTGCAAGAACTACGAT R: CAGGGGACCCTGCAATGAC	60.11	191
MDH2	F: TCCCGTAACACACAGACAGC R: GCGTTGGTGTTGAACAGGTC	59.97	117
LDHC	F: CCTTCACTGCTCACGTTTGG R: CGCCAATCCCTTTTCACCAC	58.53	218
SIRT5	F: CACCCCTCCAGCTTACCAAG R: TGTGTCCATCCTGTAATGTCGG	60.04	325

## Data Availability

The data presented in this study are available within the article and Supplementary Materials.

## Supplementary Materials

The supporting information can be downloaded at: https://www.mdpi.com/article/10.3390/ijms241310983/s1.
