# Supplementary material for: Cryopreservation Induces Acetylation of Metabolism-Related Proteins in Boar Sperm
Source: Int J Mol Sci. 2023 Jul 1;24(13):10983. doi: 10.3390/ijms241310983 (PMC10341673; doi:10.3390/ijms241310983)
Supplement: Supplementary file 1 [file ijms-24-10983-s001.zip › Supplementary Figures S1 and S2.pdf]

International Journal of *Molecular Sciences*

MDPI

Supplementary Material

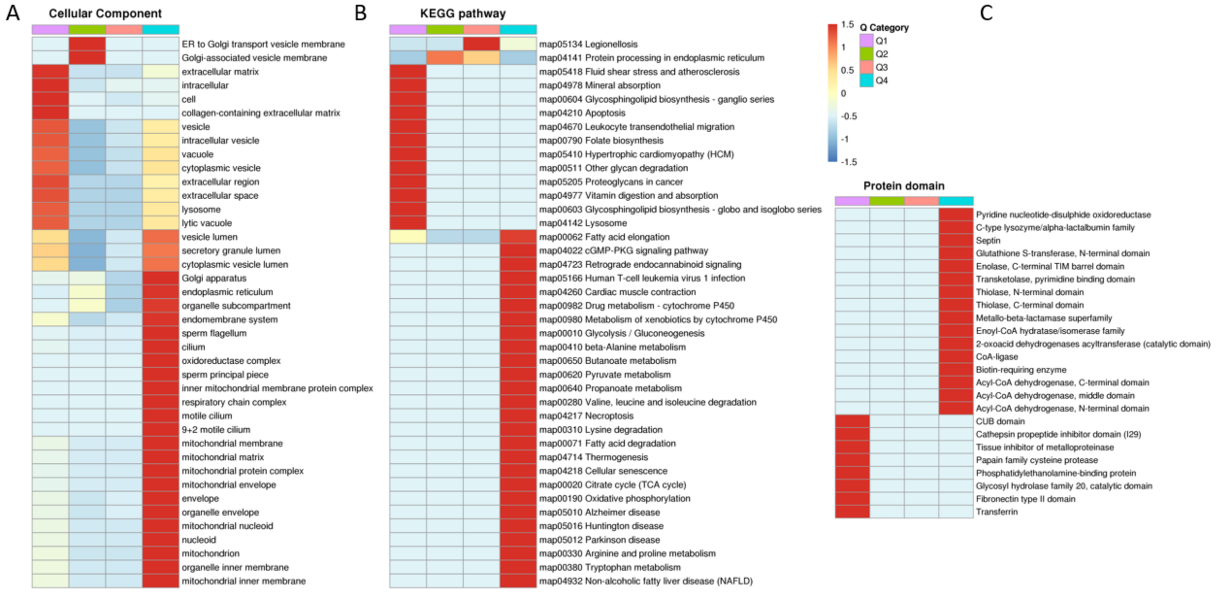

Supplementary Figure S1: Cluster analysis showing the degree of enrichment of differentially expressed proteins in fresh and frozen sperm groups. Cluster analysis using Gene Ontology (GO) for Biological Function (A); Cellular Component (B) and Molecular Function (C). Intense red color indicates highest enrichment and blue color indicates the lowest enrichment. Different groups (Q1, Q2, Q3, Q4) are represented horizontally and the functions vertically.

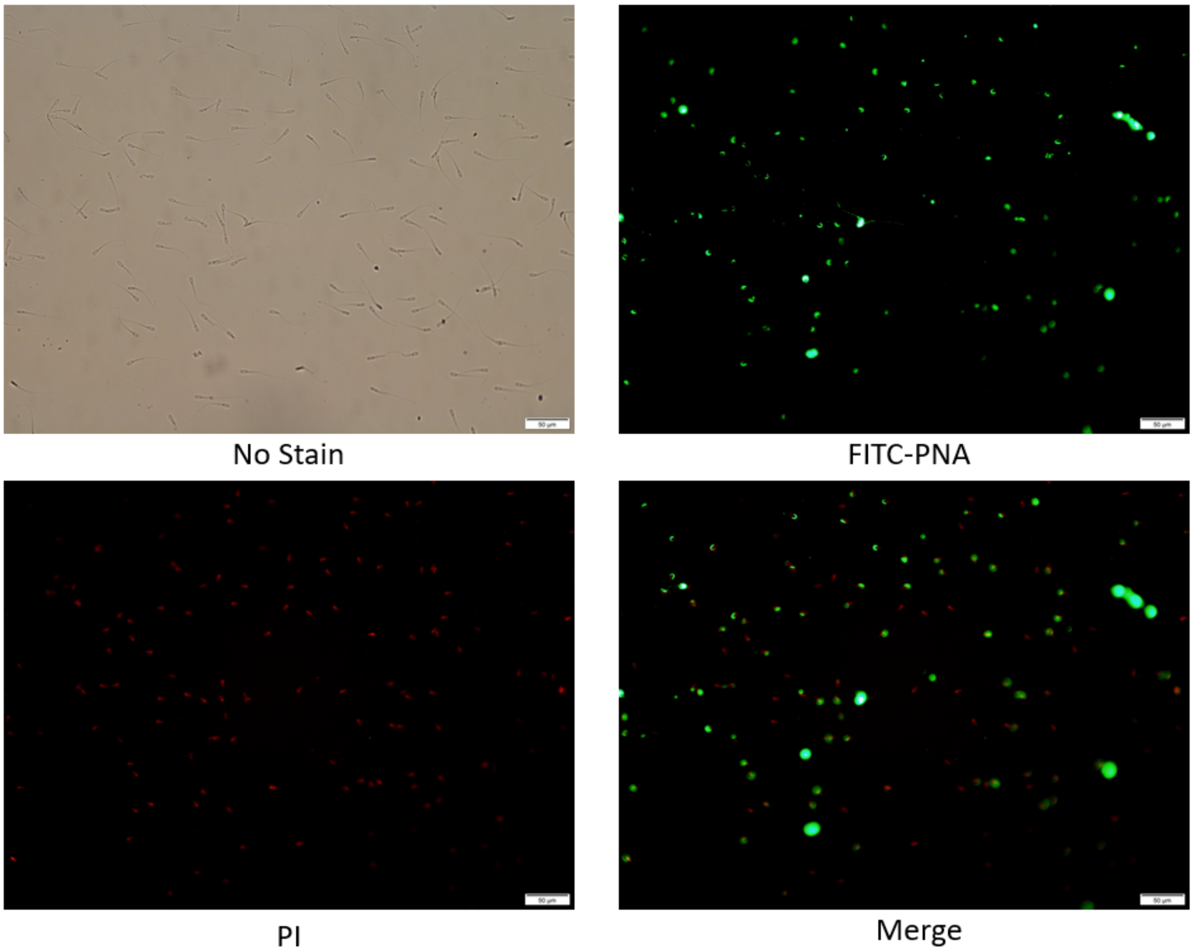

Supplementary Figure S2: Acrosomal Status of the Fresh Sperm treated with FITC-PNA and PI.
